# Childhood pneumonia and crowding, bed-sharing and nutrition: a case-control study from The Gambia

**DOI:** 10.5588/ijtld.15.0993

**Published:** 2016-10-01

**Authors:** S. R. C. Howie, J. Schellenberg, O. Chimah, R. C. Ideh, B. E. Ebruke, C. Oluwalana, G. Mackenzie, M. Jallow, M. Njie, S. Donkor, K. L. Dionisio, G. Goldberg, K. Fornace, C. Bottomley, P. C. Hill, C. C. Grant, T. Corrah, A. M. Prentice, M. Ezzati, B. M. Greenwood, P. G. Smith, R. A. Adegbola, K. Mulholland

**Affiliations:** * Medical Research Council Unit, Fajara, The Gambia; † Department of Paediatrics: Child & Youth Health, University of Auckland, Auckland; ‡ Centre for International Health, University of Otago, Dunedin, New Zealand; § London School of Hygiene & Tropical Medicine, London, UK; ¶ Child Health Department, University of Benin, Teaching Hospital, Benin City, Nigeria; # Ministry of Health and Social Welfare, Banjul, The Gambia; ** Harvard School of Public Health, Department of Global Health and Population, Boston, and Harvard School of Public Health, Department of Environmental Health, Boston, Massachusetts; †† National Exposure Research Laboratory, US Environmental Protection Agency, Research Triangle Park, North Carolina, USA; ‡‡ Medical Research Council (MRC) Human Nutrition Research, Cambridge; §§ MRC-Public Health England Centre for Environment and Health, Imperial College London, London; ¶¶ Department of Epidemiology and Biostatistics, School of Public Health, Imperial College London, London, UK; ## GlaxoSmithKline Vaccines, Wavre, Belgium; *** University of Melbourne, Parkville, New South Wales, Australia

**Keywords:** Africa, risk factors, cough, household air pollution, particulate matter

## Abstract

SETTING: Greater Banjul and Upper River Regions, The Gambia.

OBJECTIVE: To investigate tractable social, environmental and nutritional risk factors for childhood pneumonia.

DESIGN: A case-control study examining the association of crowding, household air pollution (HAP) and nutritional factors with pneumonia was undertaken in children aged 2–59 months: 458 children with severe pneumonia, defined according to the modified WHO criteria, were compared with 322 children with non-severe pneumonia, and these groups were compared to 801 neighbourhood controls. Controls were matched by age, sex, area and season.

RESULTS: Strong evidence was found of an association between bed-sharing with someone with a cough and severe pneumonia (adjusted OR [aOR] 5.1, 95%CI 3.2–8.2, *P* < 0.001) and non-severe pneumonia (aOR 7.3, 95%CI 4.1–13.1, *P* < 0.001), with 18% of severe cases estimated to be attributable to this risk factor. Malnutrition and pneumonia had clear evidence of association, which was strongest between severe malnutrition and severe pneumonia (aOR 8.7, 95%CI 4.2–17.8, *P* < 0.001). No association was found between pneumonia and individual carbon monoxide exposure as a measure of HAP.

CONCLUSION: Bed-sharing with someone with a cough is an important risk factor for severe pneumonia, and potentially tractable to intervention, while malnutrition remains an important tractable determinant.

## RESUME

CONTEXTE : Grand Banjul et régions de la Haute Rivière, Gambie.

OBJECTIF : Rechercher les facteurs de risque modifiables, sociaux, environnementaux et nutritionnels de pneumonie de l'enfant.

SCHÉMA : Une étude cas témoins examinant les associations de la promiscuité, de la pollution domestique de l'air et des facteurs nutritionnels avec la pneumonie a été entreprise chez des enfants âgés de 2 à 59 mois. Ont été comparés 458 enfants atteints de pneumonie grave, définie en fonction des critères modifiés de l'Organisation Mondiale de la Santé, à 322 enfants atteints de pneumonie non grave ; ces deux groupes ont été comparés à 801 témoins du voisinage. Ces témoins ont été appariés sur l'âge, le sexe, le quartier et la saison.

RÉSULTATS : Nous avons mis en évidence des preuves nettes d'association entre le fait de partager un lit avec quelqu'un qui tousse et une pneumonie grave (OR ajusté [aOR] 5,1 ; IC95% 3,2–8,2 ; *P* < 0,001) et une pneumonie non grave (ORa 7,3 ; IC95% 4,1–13,1 ; *P* < 0,001), 18% des cas graves étant estimés attribuables à ce facteur de risque. La malnutrition a également été liée de façon flagrante à la pneumonie, l'association étant la plus forte entre la malnutrition grave et la pneumonie grave (ORa 8,7 ; IC95% 4,2–17,8 ; *P* < 0,001). Aucune association n'a été trouvée entre la pneumonie et l'exposition individuelle au monoxyde de carbone comme mesure de pollution domestique.

CONCLUSION : Le fait de partager un lit avec une personne qui tousse est un facteur de risque important de pneumonie grave. Ce facteur est potentiellement accessible à une intervention, tandis que la malnutrition reste un facteur important mais qui peut être amélioré.

## RESUMEN

MARCO DE REFERENCIA: La Gran Zona de Banjul y la división Upper River de Gambia.

OBJETIVO: Investigar los factores de riesgo ambientales y nutricionales de aparición de neumonía en los niños que se pueden modificar.

MÉTODO: Fue este un estudio de casos y testigos que examinó la asociación del hacinamiento, la contaminación del aire doméstico y los factores nutricionales con la aparición de neumonía en los niños de edad de 2–59 meses. Se compararon 458 niños con diagnóstico de neumonía grave según los criterios modificados de la Organización Mundial de la Salud, con 322 niños con neumonía que no era grave, y estos grupos con un grupo testigo conformado por 801 niños del vecindario, apareados en función de la edad, el sexo, la región y la estación del episodio.

RESULTADOS: Se pusieron en evidencia sólidas pruebas de la asociación entre el hecho de compartir la cama con una persona que tose y la neumonía grave (OR ajustado [ORa] 5,1; IC95% 3,2–8,2; *P* < 0,001) y la neumonía que no es grave (ORa 7,3; IC95% 4,1 a 13,1; *P* < 0,001); se estimó que el 18% de los casos graves era atribuible a este factor de riesgo. La desnutrición y la neumonía exhibieron una clara asociación, que fue más fuerte entre la desnutrición grave y la neumonía grave (ORa 8,7; IC95% 4,2 a 17,8; *P* < 0,001). No se encontró ninguna correlación entre la neumonía y la exposición personal al monóxido de carbono, como una medida de contaminación ambiental domiciliaria.

CONCLUSIÓ N: El hecho de compartir la cama con una persona que tose constituye un importante factor de riesgo de padecer una neumonía grave y sería posible abordarlo con una intervención; la desnutrición sigue siendo un importante factor determinante que se debe solucionar.

PNEUMONIA is the biggest single cause of death in children globally, accounting for 15% of over 6 million deaths in children aged <5 years in 2013.^1^ Half of these deaths occur in sub-Saharan Africa, and The Gambia, like other countries in this region, suffers a high toll from pneumonia.^2–4^ The burden of death from pneumonia needs to be substantially reduced if global child survival targets are to be met.^5^

No single intervention, including vaccination against *Haemophilus influenzae* type b (Hib) and *Streptococcus pneumoniae*,^6–9^ will alone adequately address the complex challenge pneumonia presents. Rather, a multi-pronged approach encompassing vaccination, improved case management, and, importantly, social, environmental and nutritional interventions, is needed; these are likely to be as important in reducing pneumonia mortality in lowand middle-income countries as they were in pre-antibiotic America.^10^ Such an approach is likely to be cost-effective.^11^

The objective of the present study was to identify the most important of these social, environmental and nutritional factors.

Our primary exposures of interest were bed-sharing with someone with a cough, exposure to household air pollution (HAP) and feeding practices, particularly the early introduction of solids to the infant diet. Crowding is an important risk factor for a range of infectious diseases,^12^ and while pneumonia has been associated with household crowding^13–19^ it is not known if bed-sharing with someone with a cough mediates this association. While HAP, mostly from cooking smoke, is, like tobacco smoke,^20^ believed to be an important risk factor for pneumonia,^4^ few studies have directly measured exposure at the individual level. Malnutrition is associated with pneumonia; however, the role of interference with breast feeding through early introduction of solids is not clear.

## STUDY POPULATION AND METHODS

### Study setting and population

**Figure 1.**
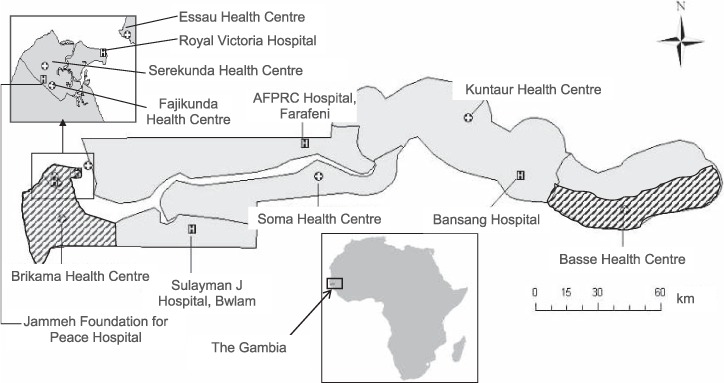
Map of The Gambia showing the study sites in the Greater Banjul and Basse areas (cross-hatched), along with hospitals and major health centres. AFPRC = Armed Forces Provisional Ruling Council.

The Gambia, a resource-poor country in West Africa with a population of 1.8 million, has high child mortality (74 children under 5 per 1000 live births) and a low human immunodeficiency virus (HIV) prevalence of <2%.^21^,^22^ The aetiology of childhood pneumonia has been historically dominated by S. pneumoniae and H. influenzae (vaccination for S. pneumoniae was introduced in 2009 and for H. influenzae type b in 1997), along with common respiratory viruses, notably respiratory syncytial virus. Immunisation coverage is high,^23^ and one third of men smoke.^24^ A case-control study of environmental and nutritional risk factors for severe childhood pneumonia was undertaken at two sites, one peri-urban (the Greater Banjul area) and the other rural (the Basse area) (Figure 1).

### Selection of participants

We conducted a prospective case-control study comparing severe pneumonia cases with children with non-severe pneumonia and community controls. Cases were children aged 2–59 months with severe pneumonia who presented to the Medical Research Council (MRC) hospital in Fajara, the Edward Francis Small Teaching Hospital (EFSTH) in Banjul, or the major health centres at Fajikunda, Serekunda, Brikama and Basse, between June 2007 and September 2010. We defined severe pneumonia using the modified World Health Organization (WHO) criteria,^25^ i.e., cough or difficulty in breathing, plus any of the following: lower chest wall indrawing, nasal flaring, or an oxygen saturation of <90% on pulse oximetry (the last defining very severe pneumonia). Non-severe pneumonia was defined as cough or difficulty in breathing plus tachypnoea, defined using WHO age-stratified cut-offs. We excluded children with a cough of ⩾2 weeks' duration, severe anaemia (Hb <6 g/dl) or wheeze on auscultation.

**Figure 2.**
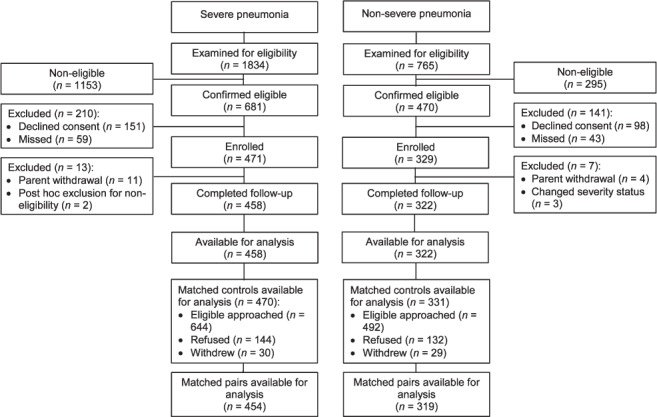
Profile of entry into the study.

We selected two comparison (control) groups. Comparison Group 1 comprised children aged 2–59 months with WHO-defined non-severe pneumonia recruited from the out-patient departments of the health facilities from which the severe pneumonia cases were recruited. We frequency-matched these children to severe cases by municipal area of residence, season, age and sex. Comparison Group 2 comprised children in the community aged 2–59 months without pneumonia, individually matched to cases by neighbourhood, season, age and sex. For each case, we selected a Comparison Group 2 community control as follows: from the compound (a collection of related dwellings usually demarcated by a fence) of the case, a fieldworker walked at least 50 paces in a direction chosen at random by spinning a pen.^26^ Then, at the nearest compound s/he identified a child by random selection among the eligible children. If consent was declined, the next randomly selected eligible child was identified, from the next compound, if necessary. Comparison Group 2 participants were brought to the clinic to be assessed by a study doctor and were later visited at home by a fieldworker in a sequence that mirrored the exposure measurements for cases. Furthermore, we selected community controls in exactly the same manner as for the children selected with non-severe pneumonia (Control Group 1), allowing examination of risk factors for non-severe pneumonia (Figure 2).

### Measurement of exposure

We measured exposures using a questionnaire administered to the primary care giver (including behaviour and fuel use); observation; examination in the field or clinic; and using a carbon monoxide (CO) diffusion tube attached to the child for HAP measurement.^27^ Particulate matter (PM) is the HAP exposure of interest; however, PM measurement devices are relatively bulky and cannot be used in small children. Exposure to fine particulate matter (PM_2.5_) was therefore modelled using directly measured household-level CO, PM and fuel data coupled with personal CO data. However, validation of the model using direct measurement of PM in a subset of ambulatory children failed through the lack of correlation between directly measured and modelled PM, rendering PM exposure estimates unreliable.^28–30^ Crowding-related questions focusing on the month before enrolment established household size, how many people slept in the child's room, where they slept and whether the child shared a bed with anyone having a cough. We measured current and past feeding practices by questionnaire and current nutritional status using anthropometry.

### Bias and confounding

We completed exposure measurements in the home as soon after enrolment as possible. We addressed possible recall bias resulting from the delay in interviewing cases until after discharge by also asking questions at the time of admission. We attempted to minimise information bias from questionnaires by careful training of interviewers, supervision in the field and by blinding interviewers to participants' disease status. Interviewees were not aware of the risk factors of most interest.

We considered potential confounders within a hierarchical framework of determinants of pneumonia.^31^ Age was a likely confounder for all associations of interest. Mothers preferentially keep infants in their own bed, and on their back during cooking, and younger children were less likely to have been weaned. We addressed confounding by age through matching and during analysis, and did the same for sex, geographical area and season, which were also a priori designated as potential confounders. We measured a range of other social, demographic and environmental exposures, including variables related to education, hygiene, access to health care and vaccination, and addressed these as possible confounders in the analysis. Similarly, we constructed an index of socio-economic status using principal components analysis.^32^,^33^

### Analysis, sample size and data management

We identified risk factors for severe and non-severe pneumonia by comparing cases to individually matched community controls; severe pneumonia cases were also compared to non-severe pneumonia cases to identify specific factors associated with severe disease. For each exposure, we constructed a logistic regression model (conditional in the case of the individually matched analyses) that included the exposure of interest, a priori confounders (age, sex, geographical area and season) and any other confounders that changed the adjusted odds ratio (aOR) by ⩾15%. *P* values were calculated using Wald tests, except for interactions, which were tested using the likelihood ratio test (LRT). We calculated the proportion of cases attributable to bed-sharing with someone with a cough (i.e., the population attributable fraction) as the product of the proportion of cases exposed to bed-sharing with someone with a cough and the attributable fraction (1-1/OR, where OR compares the odds of being a case when in a household where someone had a cough to the odds of being a case when bed-sharing with someone with a cough).

The study size—of at least 300 cases of severe pneumonia, 300 cases of non-severe pneumonia and 600 neighbourhood controls—was chosen so that there would be at least 80% power to detect an OR of 2 at the 5% significance level for exposures with a prevalence of between 10% and 80%. Data were double-entered and verified using an SQL database (Microsoft Corporation, Redmond, WA, USA). Analyses were performed using Stata versions 11 and 12 (StataCorp, College Station, TX, USA).

### Ethics

We obtained written informed consent for participation in the study from the parents or legal guardians of the cases and controls. The study was approved by the Gambian Government-Medical Research Council Joint Ethics Committee, Banjul, The Gambia, and the Ethics Committee of the London School of Hygiene & Tropical Medicine, London, UK (SCC/EC1062).

## RESULTS

### Study participants

A total of 458 severe pneumonia cases, 322 non-severe pneumonia cases and 801 community controls were available for analysis (Figure 2). Of 681 patients, 458 (67%) severe pneumonia cases identified initially as eligible were included in the analyses, along with 69% (322/470) of non-severe pneumonia cases and 71% (801/1136) of community controls. Eligible non-participants were similar to participants with respect to age, sex, area of residence and season of enrolment, with the exceptions that community control non-participation was more likely in Greater Banjul residents and among those invited to participate in the rainy season, at which time non-severe pneumonia case non-participation was also more likely, probably because of the pressure to sow and reap crops and adverse road conditions. The demographic, social and environmental characteristics of the three study groups were similar (Table 1). The prevalence of exposures of interest is shown in Tables 2 and 3. aORs for all exposures of interest in the study were generated, including age, sex, season and location in the final regression models, with few additional confounders being identified (see Tables 4 and 5).

**Table 1:**
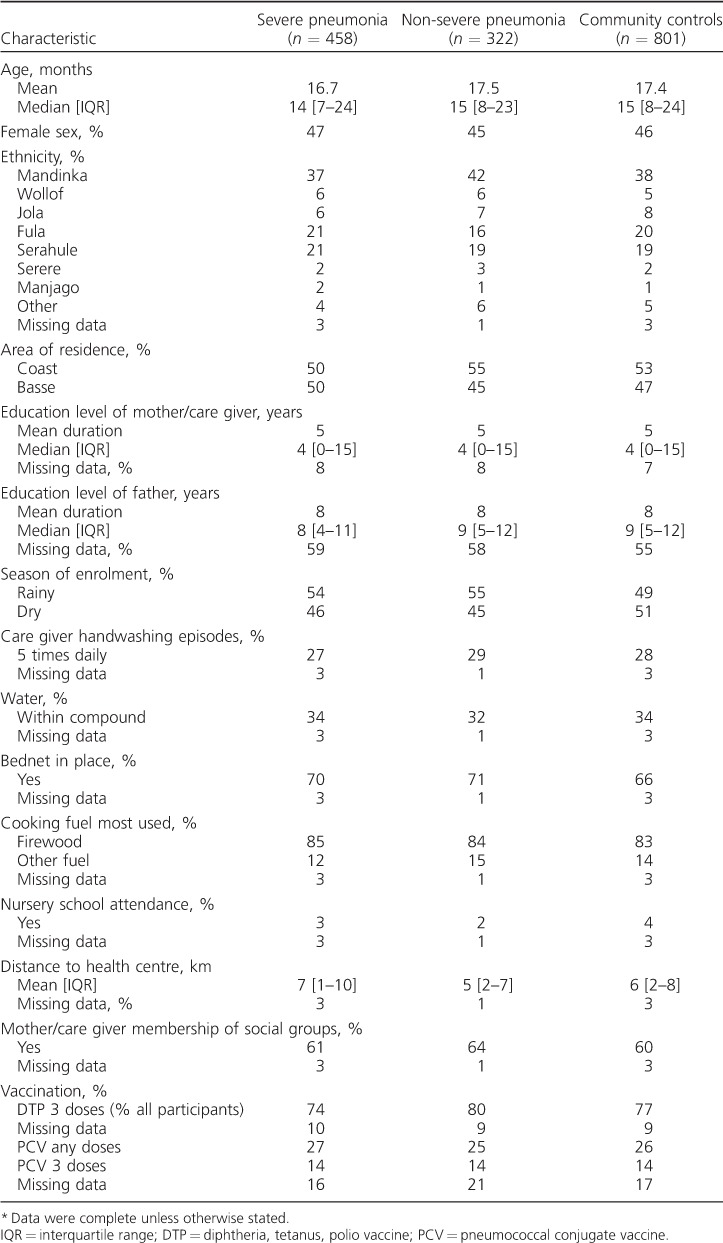
Characteristics of the study participants ^*^

**Table 2:**
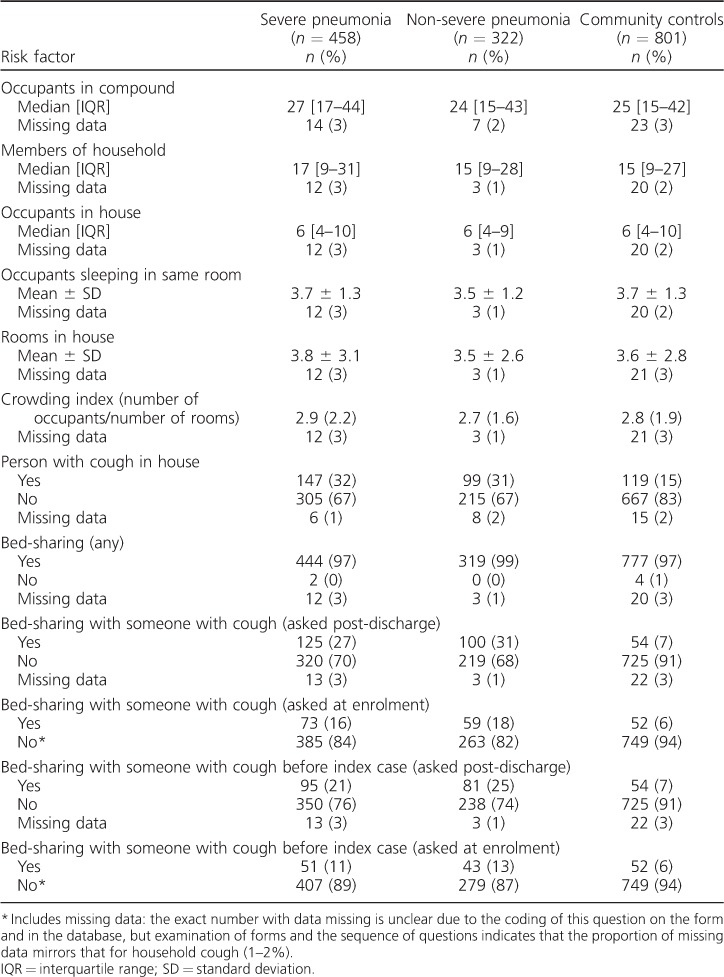
Frequency and prevalence of crowding-related exposures by severity category

**Table 3:**
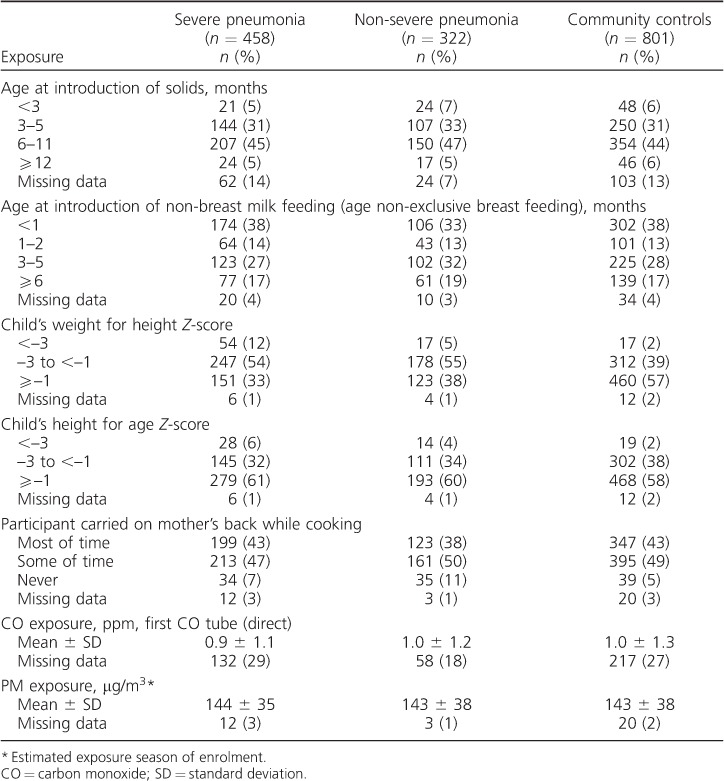
Frequency and prevalence of nutrition and household air pollution-related exposure variables by participant category

**Table 4:**
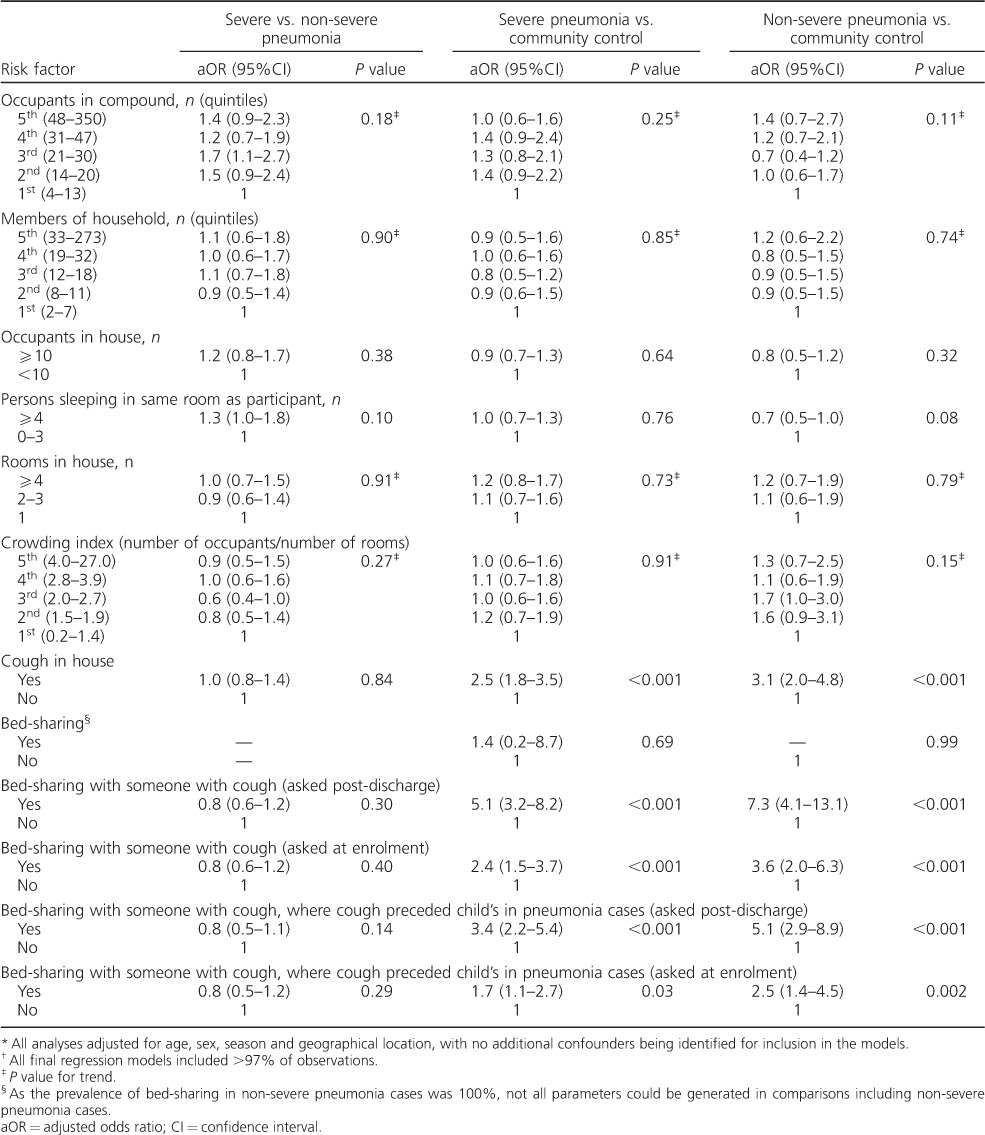
aORs ^*^ for the association between key crowding-related exposures and severe pneumonia and non-severe pneumonia ^†^

**Table 5:**
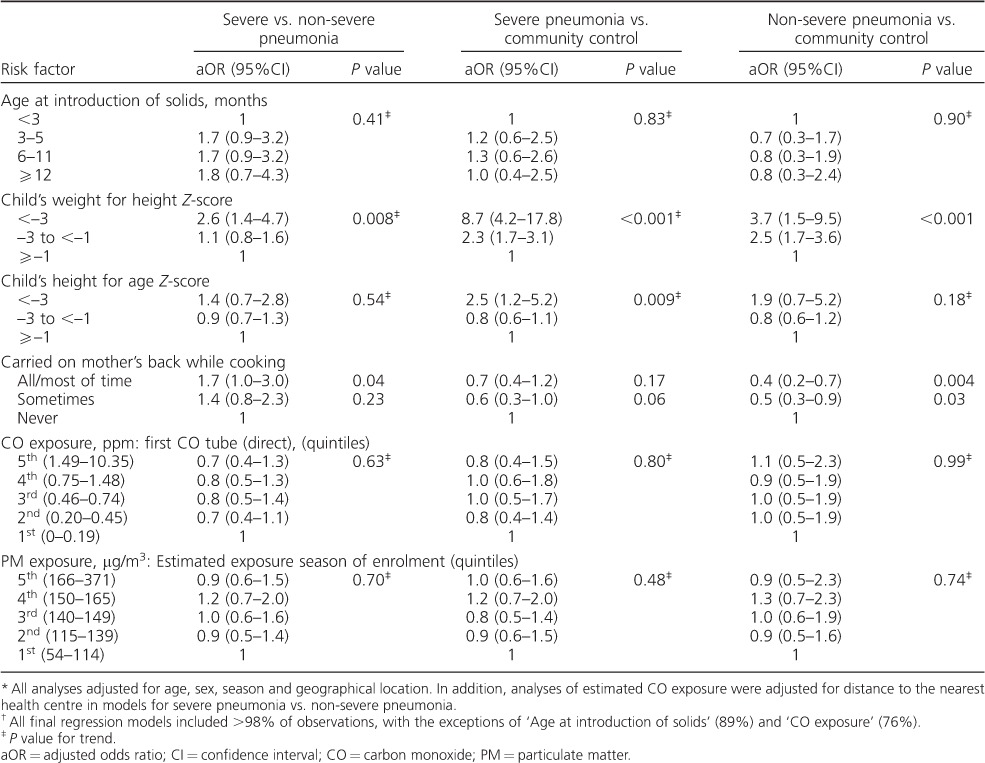
aORs ^*^ for the association between key nutritional and household air pollution exposures of interest and severe pneumonia and non-severe pneumonia ^†^

### Association of crowding and bed-sharing with pneumonia

Findings related to crowding and bed-sharing are shown in Table 2, Appendix Table A and Table 4.^*^ There was no consistent evidence of associations between the number of people in the compound, household or house and pneumonia (severe or non-severe). There was also little evidence of an association between pneumonia and the number of people sleeping in the same room as the sick child.

Having someone with cough in the house was associated with severe pneumonia (compared to community controls, aOR 2.5, 95%CI 1.8–3.5, *P* < 0.001) and non-severe pneumonia (aOR 3.1, 95%CI 2.0–4.8, *P* < 0.001). There was strong evidence of associations between bed-sharing with someone with a cough and both severe pneumonia (aOR 5.1, 95%CI 3.2–8.2, *P* < 0.001) and non-severe pneumonia (aOR 7.3, 95%CI 4.1–13.1, *P* < 0.001) in comparison with community controls.

The OR between bed-sharing with someone with a cough (asked after discharge) and severe pneumonia was stronger for cases from Basse (aOR 10.2, 95%CI 4.4–24.0, *P* < 0.001) than for cases from Greater Banjul (aOR 3.4, 95%CI 1.9–6.2, *P* < 0.001) (*P* value for the difference = 0.03).

There was strong evidence of a dose-response relationship between pneumonia and a child's exposure to someone with cough across a gradient of exposure: for severe pneumonia (compared to community controls), the aOR was 2.3 (95%CI 1.6–3.4, *P* < 0.001) for someone with cough in the household (but not in the same bed) and 6.2 (95%CI 3.8–10.1, *P* < 0.001) for someone with cough in the same bed. In households with someone with cough, the aOR for the effect of a bedmate with cough and severe pneumonia was 2.7 (95%CI 1.6–4.7, *P* < 0.001).

Among cases of severe pneumonia in households with someone with cough, the prevalence of bed-sharing with someone with cough was 28%; and, assuming causality, the proportion of all severe cases attributable to this exposure was estimated to be 18%.

### Association of malnutrition with pneumonia

Findings related to nutrition and cooking smoke-related risk factors are shown in Tables 3 and 5. No association was found between early introduction of solids or other forms of mixed feeding and severe pneumonia. However, strong evidence was found of an association between malnutrition and both severe and non-severe pneumonia. For severe pneumonia and severe malnutrition (weight-for-height *Z*-score [WHZ] < −3), the aOR was 8.7 (95%CI 4.2–17.8, *P* < 0.001), and for severe pneumonia and less severe malnutrition (WHZ −1to < −3) the aOR was 2.3 (95%CI 1.7–3.1, *P* < 0.001). There was evidence also of an association between severe stunting (height-forage *Z*-score < −3) and severe pneumonia (aOR 2.5, 95%CI 1.2–5.2, *P* = 0.014).

### Association of cooking smoke exposure with pneumonia

Firewood was the predominant fuel in 84% of households. Mixed evidence was found concerning back-carrying during cooking. Weak evidence was found for an association between being carried on the mother's back while cooking and severe pneumonia compared to non-severe pneumonia (aOR 1.7, 95%CI 1.0–3.0, *P* = 0.04); however, in comparison with controls there was evidence of a lower risk of non-severe pneumonia (aOR 0.4, 95%CI 0.2–0.7, *P* = 0.004). No associations were found between pneumonia and measured CO exposure or modelled PM exposure (Tables 3 and 5).

## DISCUSSION

We found consistent evidence of an association between bed-sharing with someone with cough and both severe and non-severe pneumonia. The relationship was moderately strong, with aORs ranging from 1.7 to 7.3, showed a dose-response relationship, and was consistently statistically significant. There was no evidence of differential risks for severe and non-severe pneumonia; thus, bed-sharing with someone with cough was not associated with more severe disease among those who had pneumonia. No association was seen with mixed feeding (breast milk and other sources) in this highly breast-fed population, but a strong association between malnutrition and pneumonia was observed. The technical limitations still associated with measurement of individual-level HAP exposure, specifically the inability to directly measure PM (the exposure of interest) at the individual level in young children, and the lack of correlation between CO (measurable at individual level) and PM hampering modelled estimates, make the findings of no association between pneumonia and PM difficult to interpret.

The similar risks between bed-sharing with someone with cough for non-severe and severe pneumonia may be explained in more than one way. The level of exposure to the infecting organism may not be a dominant factor determining the severity of disease, or it may be due to overlap between the severe and non-severe disease phenotypes. The clinical features of these two groups showed apparent differences in measures such as history of difficulty in breathing (89% vs. 49%) and lethargy (17% vs. 0%); however, it is possible that there was insufficient difference between the phenotypes for a study of this size to demonstrate differences between the groups for this exposure.

The lack of evidence for risks associated with non-exclusive breast-feeding in this almost universally breast-fed population is consistent with previous studies in The Gambia,^16^,^34^ but contrasts with evidence from other settings.^35^ This may be due to non-breast milk contributing relatively little to the nutrition of mixed-fed children in the Gambian setting. The design of the present study, with its risk of recall bias for this exposure, did not allow examination of the high-risk newborn period in which early initiation of breast feeding appears highly protective.^36^

There was strong evidence for a graded association between malnutrition and both severe and non-severe pneumonia. While malnutrition may be worsened by acute illness, the short duration of the illness in cases enrolled in this study (median 3 days) and the lack of evidence of dehydration, which can lead to overestimation of malnutrition,^37^ suggest that a substantial portion of the observed malnutrition preceded the illness. The association observed between stunting and severe pneumonia also supports the conclusion that previous nutritional status increased the risk of pneumonia. This is consistent with the broad evidence that malnutrition is a tractable risk factor amenable to practical interventions, and that these must be promoted.^38^

A number of studies have addressed the issue of crowding as a risk factor for pneumonia. Attendance at a day-care centre has been shown to increase the risk of pneumonia.^14^ There is evidence from several countries that crowding at the household level is also a risk factor for pneumonia across a spectrum of severity. A case-control study from Brazil showed an association between household overcrowding and death from pneumonia in infants.^15^ Another case-control study from India showed an association between severe pneumonia and sharing a bedroom,^19^ and a cohort study from Kilifi, Kenya, showed a modest association between crowding (number and proximity of siblings) and all-cause pneumonia.^17^ A 1993 Gambian study of risk factors for pneumonia mortality under 2 years of age^13^ found no association with the number of co-occupants in the child's room or in their bed, while another Gambian study found no association between bedroom co-occupancy and pneumococcal disease.^16^ A protective association between bed co-occupants and pneumonia was observed in the multi-country BOSTID (Board on Science and Technology for International Development) study.^18^

The lack of evidence in this study of a consistent association between pneumonia and the numbers of occupants in the compound, household or house, and with the density of occupation within the house, can be explained in two ways: either general crowding is not a crucial factor in the development or severity of pneumonia in this context, or such crowding is so uniform that a case-control study is unable to identify it as an important contributor to overall pneumonia risk. Bedroom co-occupancy was not identified as a risk factor in this study, and this is consistent with previous Gambian studies,^13^,^16^ but differs from the Indian study^19^ and the Kenyan study^17^ noted above. The authors are not aware of previous studies specifically examining bed-sharing with someone with cough.

The association observed in this study between pneumonia and bed-sharing with someone with cough is strong and consistent, shows a dose-response relationship and is biologically plausible, supporting the conclusion that this association is real. The possibility that this finding is subject to bias, confounding, random error or a combination of these should also be considered. Although care was taken to minimise selection and information bias, residual bias is likely. Participation rates of eligible children were around 70%, leaving room for bias, despite the apparent similarity of participants and non-participants. Having acknowledged these limitations, the observed association appears robust in our study.

Assuming causality, we estimate that 18% of severe pneumonia cases are attributable to bed-sharing with someone with cough, which indicates the potential public health importance of this risk factor. The feasibility of developing an intervention to reduce the exposure of children to a bedmate with cough therefore needs to be considered. A direct health education message to avoid putting a child in the bed of a person with cough is the most obvious route to follow, but there may be other ways of achieving this goal. Designing any intervention of this kind would need a sound background knowledge of the sociological dynamics of the household and community, and poses substantial challenges. Nevertheless, such an intervention, once developed, could have a substantial public health impact.

In conclusion, our study suggests that bed-sharing with someone with cough is an important risk factor for severe pneumonia in young children. Further work to design and test an appropriate intervention is required. The study also suggests that malnutrition remains an important tractable determinant for pneumonia.

## Figures and Tables

**Table A:**
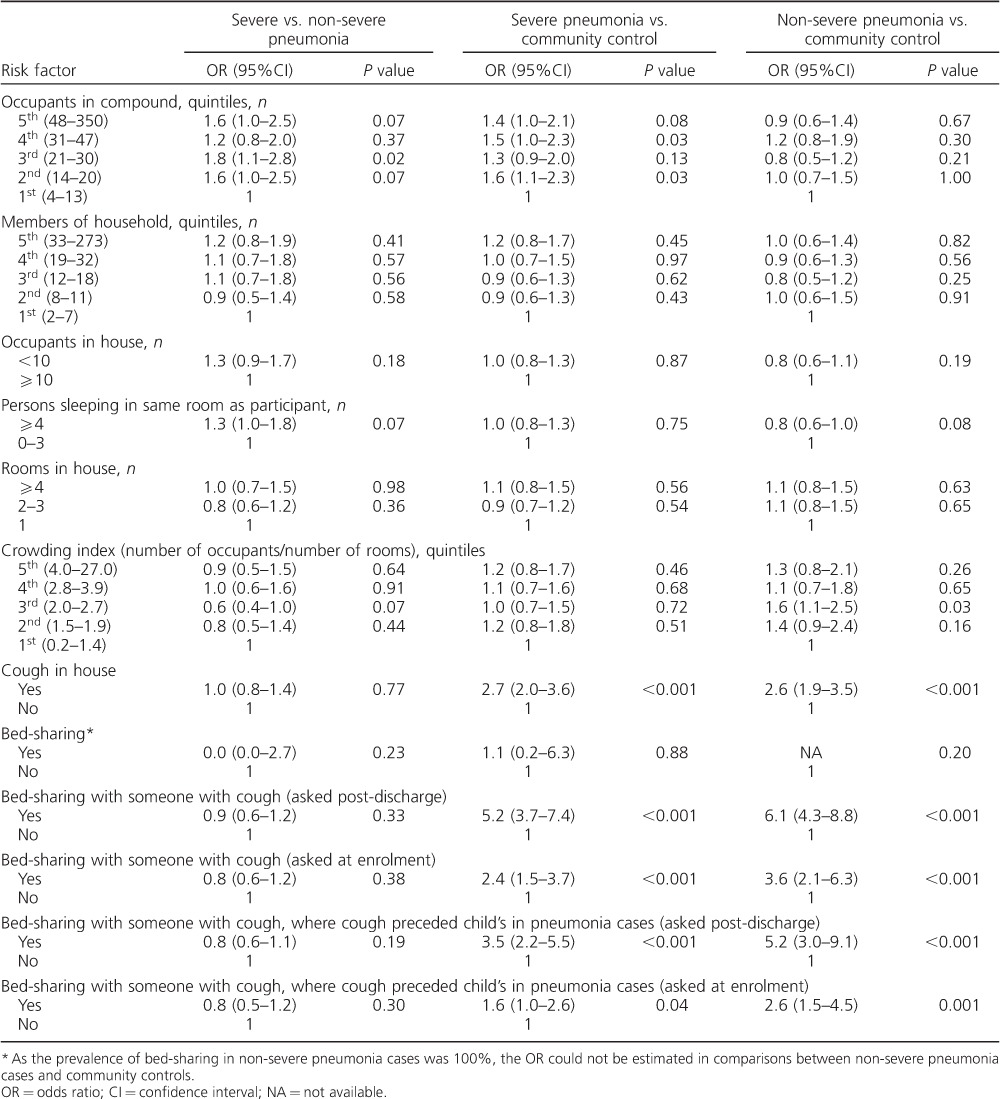
Univariate analysis of crude ORs for the association of crowding-related exposures with severe pneumonia and non-severe pneumonia.

## Footnotes

^*^ The appendix is available in the online version of this article, at http://www.ingentaconnect.com/content/iuatld/ijtld/2016/00000020/000000010/art00026
